# Effects of *Lactiplantibacillus plantarum* and Cellulase Inoculation on Silage Quality of Grape Branches and Leaves

**DOI:** 10.3390/microorganisms13122842

**Published:** 2025-12-14

**Authors:** Changhao Li, Zhiwei Huo, Shuangming Li, Rongzheng Huang, Yingli Ji, Chunhui Ma, Shaoqi Cao, Fanfan Zhang

**Affiliations:** 1 College of Animal Science & Technology, Shihezi University, Shihezi 832000, China; 15588213165@163.com (C.L.); h69896882@163.com (Z.H.); lishuangming0410@163.com (S.L.); huangrz2013@163.com (R.H.); chunhuima@126.com (C.M.); 2 Bingtuan Key Laboratory for Efficient Utilization of Non-Grain Feed Resources, Shihezi 832000, China; 3 Kashgar Prefecture Animal Husbandry and Veterinary Bureau of Xinjiang Uygur Autonomous Region, Kashi 844000, China; 4 Zhijiang Animal Husbandry and Veterinary Service Center, Zhijiang 443000, China; 5 Xinjiang Production and Construction Corps Animal Husbandry and Veterinary Work Station, Urumqi 830000, China; jyl050293@163.com; 6 Xinjiang Uygur Autonomous Region Animal Husbandry Station, Urumqi 830000, China

**Keywords:** grape by-products, silage additive, unconventional feed, fiber degradation

## Abstract

To tackle grape branch and leaf waste and alleviate global feed shortages, this study tested silage made from Xinjiang ‘Seedless White’ grape foliage. Three treatments were established: CK (control, only grape branches and leaves), PL (inoculated with 5 × 10^6^ CFU·g^−1^ fresh weight *Lactiplantibacillus plantarum*), and PLC (inoculated with 5 × 10^6^ CFU·g^−1^ *L. plantarum* and 0.3% cellulase). Silages were fermented at 18–23 °C and analyzed on days 7, 15, 30, and 60. PLC reduced dry matter loss in the late fermentation stage, while lowering Neutral detergent fiber (NDF) and Acid detergent fiber (ADF) contents to solve the high-fiber issue of grape foliage silage. It also maintained a lower pH in the mid-to-late stage and higher Lactic acid (LA) content to ensure anti-spoilage. Microbiologically, PLC had the highest *Lactiplantibacillus* abundance on day 7; on day 60, its Simpson index was higher, meaning stronger microbial community stability. *Firmicutes* replaced *Cyanobacteria* as the new dominant phylum, with *Lactiplantibacillus* remaining the absolute dominant genus, and the growth of molds and yeasts was effectively inhibited. In conclusion, the combined application of *L. plantarum* and cellulase enhances the quality of grape branch and leaf silage. This study turns low-value grape branches and leaves into high-quality feed, providing support for grape branch and leaf resource utilization and helping alleviate global feed shortages.

## 1. Introduction

With the expansion and intensification of animal husbandry, natural grasslands and conventional feed resources are increasingly insufficient to meet demand [1], highlighting the need for alternative feed sources. Utilizing unconventional resources such as fruit tree branches and leaves for novel feed development is therefore of considerable importance. The grape (*Vitis vinifera* L.) belongs to the genus Vitis of the Vitaceae family and is a woody vine plant. It is one of the important fruit trees in the world and is widely cultivated in various countries and regions around the world. Grapes are not only rich in nutrients such as protein, sugars, organic acids, vitamins, and minerals, but also possess health-promoting properties, including antioxidant, antibacterial, anti-inflammatory, and cardiovascular protective effects [2]. Similarly, grape branches and leaves are rich in protein and polyphenols, sharing beneficial characteristics and presenting potential as high-quality feed ingredients [3].

According to the statistics of the Food and Agriculture Organization of the United Nations (FAO), the global grape planting area was 6.926 million hectares in 2019, and the total output reached 77 million tons. China is a major global grape producer, ranking first in output and second in planting area worldwide [4]. A large quantity of residues, including grape skins, seeds, and pruned branches and leaves, is generated during grape processing and winemaking, with an estimated annual yield of 9.42–11.78 million tons. Currently, most of these residues are disposed of via landfilling, incineration, or direct returning to fields; such practices not only cause severe resource waste but also trigger potential ecological problems, such as soil and air pollution [5]. Therefore, developing effective methods to utilize discarded post-topping grape branches and leaves as unconventional feed has become an urgent task.

Silage fermentation is an effective technique for preserving high-moisture plant materials and retaining nutrients [6]. Through anaerobic microbial fermentation, organic acids are produced that inhibit pathogenic microorganisms, reduce nutrient loss, lower crude fiber content, and improve forage palatability [7]. However, grape branches and leaves have low soluble sugar and high fiber content, making them prone to slow fermentation or spoilage during ensiling, which results in considerable nutrient loss [8]. Thus, the choice of additives is critical to fermentation quality [9,10]. *Lactiplantibacillus plantarum* and cellulase are commonly used silage additives. *L. plantarum*, an anaerobic lactic acid bacterium, ferments carbohydrates to rapidly reduce silage pH. This acidic environment inhibits molds and spoilage microorganisms, extends shelf life, and can improve forage digestibility and animal immunity through the secretion of vitamins, antimicrobial compounds, and enzymes [11].

As a compound enzyme, cellulase also plays a key role in grape branch and leaf silage [12]. Grape branches and leaves contain a relatively high amount of structural carbohydrates (such as cellulose and hemicellulose) [13]. When directly ensiled, the soluble sugar available to *Lactiplantibacillus* is insufficient, which can easily lead to slow fermentation. Cellulase can hydrolyze the structural carbohydrates in the plant cell walls, release more soluble sugars, and provide sufficient fermentation substrates for *L. plantarum* [14]. While *L. plantarum* alone has been shown to improve silage quality in certain forages, the combined effects of *L. plantarum* and cellulase on fermentation quality and microbial community dynamics in grape branch and leaf silage remain unclear.

Based on the challenges of grape branch and leaf silage and the functions of *L. plantarum* and cellulase, we hypothesize that their synergistic inoculation improves silage quality, accelerating acid production, reducing fiber, and inhibiting harmful microbes to support converting grape branches and leaves into high-quality unconventional animal feed.

## 2. Materials and Methods

### 2.1. Test Materials

Discarded branches and leaves of the fresh table grape cultivar ‘Seedless White’ grown in the experimental field of Turpan City Agricultural Science Research Institute, Xinjiang, after topping, were selected as raw materials for silage. (E 89°11′53″, N 42°56′31″, altitude 23 m). The region has a continental warm temperate desert climate, with annual sunshine of 3000–3200 h, an average annual temperature of 13.9 °C, summer extreme highs up to 49.6 °C, and surface temperatures often exceeding 70 °C. The diurnal and annual temperature variations are considerable, with an annual accumulated temperature ≥ 10 °C of over 5300 °C, a frost-free period of about 210 days, and average annual precipitation of only 16.4 mm. The raw materials were chopped into approximately 1 cm pieces before ensiling. *L. plantarum* strain was obtained from CICC (China Center of Industrial Culture Collection, Beijing, China) 23120, and cellulase was obtained from (Cellulase AP3 from Trichoderma reesei, Solarbio Science & Technology Co., Ltd., Beijing, China; Product No. C8140). The nutritional composition and microbial counts of the raw materials are shown in Table 1.

### 2.2. Experimental Design

Before ensiling, spray the raw materials with 1% fresh urea by weight. Three treatments were established: CK (control, only grape branches and leaves), PL (inoculated with 5 × 10^6^ CFU·g^−1^ *L. plantarum*), and PLC (inoculated with 5 × 10^6^ CFU·g^−1^ *L. plantarum* and 0.3% cellulase). Each bag weighs 1.5 kg of material and is vacuum-sealed in a silage plastic bag with a one-way valve (16 cm × 25 cm). Store at 18–23 °C for natural fermentation. Each treatment repeats 20 bags, for a total of 60 samples. A completely random design was adopted, with four sampling time points (7, 15, 30, and 60 days). On each sampling day, 5 bags were randomly selected from each treatment for nutritional, fermentation parameter and microbial count analysis. Microbial diversity sequencing was performed on the samples treated with CK, PL and PLC on days 0, 7 and 60.

### 2.3. Measurement Indicators and Methods

Inoculation Procedure: Test strains were cultured in MRS liquid medium and M17 liquid medium, respectively. After culturing, the concentration of strains was determined via plate counting, and the strains were applied to the silage raw materials based on the fresh weight of the materials. For the control treatment, an equivalent volume of sterile water was added instead of the strain suspension.

Chemical Analysis: Approximately 100 g of raw material or silage sample was dried at 65 °C to constant weight to determine the dry matter (DM) content [15]. The dried samples were ground using a plant grinder (DFY-1000D, Zhejiang Linda Instrument Co., Ltd., Ruian, China) and stored for subsequent analysis. Neutral detergent fiber (NDF) and acid detergent fiber (ADF) were determined by the Van Soest method [16]. Crude protein (CP) was measured by the Kjeldahl method, and ether extract (EE) was determined by Soxhlet extraction [17]. The pH value of the samples was measured using a pH meter (pH-3c, Shanghai Instrument & Electrical Scientific Instrument Co., Ltd., Shanghai, China). Ammonia nitrogen (AN) content was determined by the phenol-sodium hypochlorite colorimetric method [18]. Lactic acid (LA), acetic acid (AA), propionic acid (PA), and butyric acid (BA) were analyzed via liquid chromatography, using a C18 column (150 mm × 4.6 mm, FMF-5559-EONU, FLM Scientific Instrument Co., Ltd., Guangzhou, China). Water-soluble carbohydrate (WSC) content was measured by the anthrone colorimetric method [19].

Microbiological Analysis: Culture media and physiological saline were sterilized at 121 °C for 15 min. For microbial isolation, 10 g of silage sample was mixed with 90 mL of sterile saline in a conical flask, shaken at 150 revolutions per minute (rpm) for 30 min, and then serially diluted under a laminar flow hood.

Lactic acid bacteria (LAB) were cultured on MRS agar at 28 °C for 24 h; molds were cultured on high-salt potato dextrose agar (PDA) at 37 °C for 72 h; yeasts were cultured on malt extract agar at 37 °C for 72 h; and aerobic bacteria were cultured on nutrient agar at 28 °C for 24 h. After incubation, microbial colonies were counted to determine the population of each microbial group [20].

Bacterial Community Analysis: Genomic DNA of bacterial communities in samples was extracted using the E.Z.N.A.^®^ soil DNA kit (Omega Bio-tek, Inc., Norcross, GA, USA). The quality of extracted DNA was assessed by 1% agarose gel electrophoresis, and the concentration and purity of DNA were determined using an ultramicro spectrophotometer (Nanodrop 2000, Thermo Fisher Scientific, Inc., Waltham, MA, USA).

The V3–V4 hypervariable region of the bacterial 16S rRNA gene was amplified via polymerase chain reaction (PCR) using the primers 338F (5′-ACTCCTACGGGAGGCAGCAG-3′) and 806R (5′-GGACTACHVGGGTWTCTAAT-3′). PCR products were purified, and their quality was checked by 2% agarose gel electrophoresis. Purified PCR products were used to construct sequencing libraries, which were then sequenced on the Illumina platform for high-throughput analysis [21,22].

### 2.4. Data Processing and Analysis

Data were first collated using Microsoft Office Excel 2021. This experiment adopted a 3 × 4 factorial design, with “treatment” (3 levels: CK, PL, PLC) and “ensiling time” (4 levels: 7, 15, 30, and 60 days) as the two factors—consistent with the variables and results presented in the study. For normally distributed data (including dry matter, neutral detergent fiber, crude protein, pH, organic acids (lactic acid, acetic acid, propionic acid, butyric acid), and water-soluble carbohydrate), two-way analysis of variance (ANOVA) was performed using IBM SPSS (version 17.0; SPSS Inc., Chicago, IL, USA), followed by Duncan’s multiple comparison test to identify group differences; data are presented as the mean ± standard error (SE), with significance set at *p* < 0.05. For non-normally distributed data (microbial counts: lactic acid bacteria, yeast, mold, and aerobic bacteria), two statistical approaches were applied: first, data were log_10_-transformed to approximate normality and then analyzed via the aforementioned two-way ANOVA and Duncan’s test; second, to ensure statistical rigor, generalized linear modeling (GLM) was additionally conducted, with the data distribution specified as Poisson (for count data with small values) or lognormal (for count data with large variability) to address residual normality issues. Sequencing data of bacterial communities were analyzed via the free online Majorbio Cloud Platform (www.majorbio.com, accessed on 25 July 2025).

A GLM with Poisson distribution was used, as the Poisson distribution is suitable for discrete count data with low values and small variability. The mathematical model is defined as:

log(λijk) = μ + Ti + Sj + (T × S)ij Yijk ~ Poisson(λijk)

A GLM with lognormal distribution was used, as lognormal distribution is appropriate for count data with large values, high variability, and right-skewed distribution. The mathematical model is defined as:

log(Yijk) = μ + Ti + Sj + (T × S)ij + εijk εijk ~ N(0, σ^2^)

where Yijk is the observed microbial count for the k-th replicate in the i-th treatment and j-th ensiling time; λijk is the expected mean count of the microbial group with λijk = E(Yijk); log(λijk) is the natural logarithm link function ensuring λijk > 0; μ, Ti, Sj, (T × S)ij have the same definitions as the normal distribution model; and Yijk is assumed to follow a Poisson distribution with mean λijk.

## 3. Results

### 3.1. The Changes in Nutritional Quality During the Silage and Fermentation Process of Grape Branches and Leaves

Dynamic changes in the chemical composition of the grape branch and leaf silage are shown in Table 2. DM content in the inoculated treatments was significantly higher than in CK (*p* < 0.05), with PLC being significantly higher than PL on days 30 and 60 (*p* < 0.05). CP content in the inoculated treatments was significantly higher than CK before day 30 (*p* < 0.05). EE content showed no significant differences among treatments but varied over time, peaking on day 15 (*p* < 0.05). WSC content declined continuously, with PLC being significantly higher than CK on day 7 (*p* < 0.05). NDF and ADF contents decreased significantly over time in all treatments, with lower values in the inoculated treatments than in CK. In the early stage, NDF content in PLC was significantly lower than in PL and CK.

### 3.2. The Changes in Fermentation Quality During the Silage Process of Grape Branches and Leaves

**Figure 1:**
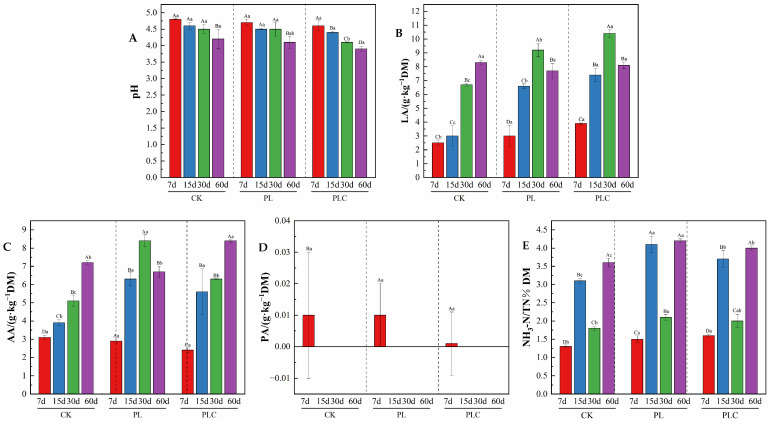
(**A**) The content of pH at different stages under various treatments; (**B**) The content of LA at different periods under various treatments; (**C**) The content of AA at different periods under various treatments; (**D**) The content of PA at different stages under various treatments; (**E**) The content of NH3-N/TN at different periods under various treatments. CK was the control group, PL was added with *L. plantarum*, and PLC was added with both *L. plantarum* and cellulase. The following table is the same. Capital letters (such as A, B, C, D) indicate that there is a statistically significant difference in the number of fermentation days under the same treatment. Lowercase letters (such as a, b, c) indicate significant differences among the groups on the day of fermentation.

Fermentation characteristics are shown in Figure 1. pH decreased over time in all treatments (*p* < 0.05), with PLC being significantly lower than CK and PL in the mid to late stages (*p* < 0.05). LA content increased then decreased, with inoculated treatments having significantly higher levels than CK on days 15 and 30 (*p* < 0.05). On day 60, AA content in PLC was significantly higher than in CK (*p* < 0.05). PA content was low and only detected in some treatments after day 15. The NH_3_-N/TN ratio fluctuated but generally increased, with PL being significantly higher than PLC for most of the time and PLC being higher than CK (*p* < 0.05).

### 3.3. The Changes in the Number of Microorganisms During the Silage Fermentation Process of Grape Branches and Leaves

Microbial population dynamics are shown in Table 3. Counts of LAB, yeasts, and aerobic bacteria decreased over time. On days 30 and 60, LAB counts in PL were significantly higher than in CK and PLC (*p* < 0.05). Yeast counts in CK were significantly higher than in PLC on days 7, 30, and 60 (*p* < 0.05). For most of the ensiling period, PLC significantly inhibited molds compared to CK (*p* < 0.05). Aerobic bacteria counts in CK and PL were mostly higher than in PLC (*p* < 0.05). Both PL and PLC inhibited yeasts, molds, and aerobic bacteria, with PLC showing more prominent inhibition, especially of molds and yeasts in later stages (*p* < 0.05).

### 3.4. Analyzing the Diversity of Grape Leaf Silage (Alpha and Beta Diversity Analysis)

**Figure 2:**
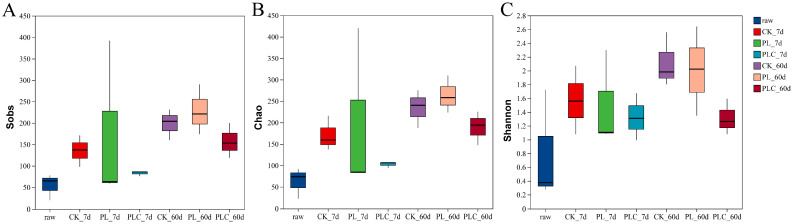
(**A**) Sobs index; (**B**) Chao Index; (**C**) Shannon index. Alpha Diversity Analysis of Grape Branch and Leaf Silage. Note: PL is the addition amount of *L. plantarum* (5 × 10^6^ CFU·g^−1^ fresh weight). PLC is the addition amount of *L. plantarum* (5 × 10^6^ CFU·g^−1^ fresh weight) and cellulase (0.3% fresh weight, enzyme activity ≥ 5000 U·g^−1^); Raw, raw materials.

Alpha diversity analysis was performed on the raw materials and silage samples after 7 and 60 days of fermentation (Figure 2). The Coverage index for all samples was close to 1 (ranging from 0.9987 to 0.9997), indicating that the sequencing depth was sufficient to capture the majority of microbial community information.

No significant differences were observed in the Shannon, Simpson, Ace, and Chao indices among the CK, PL, and PLC treatments at 7 days of fermentation. The Shannon, Ace, and Chao indices, however, showed no significant differences at this stage.

**Figure 3:**
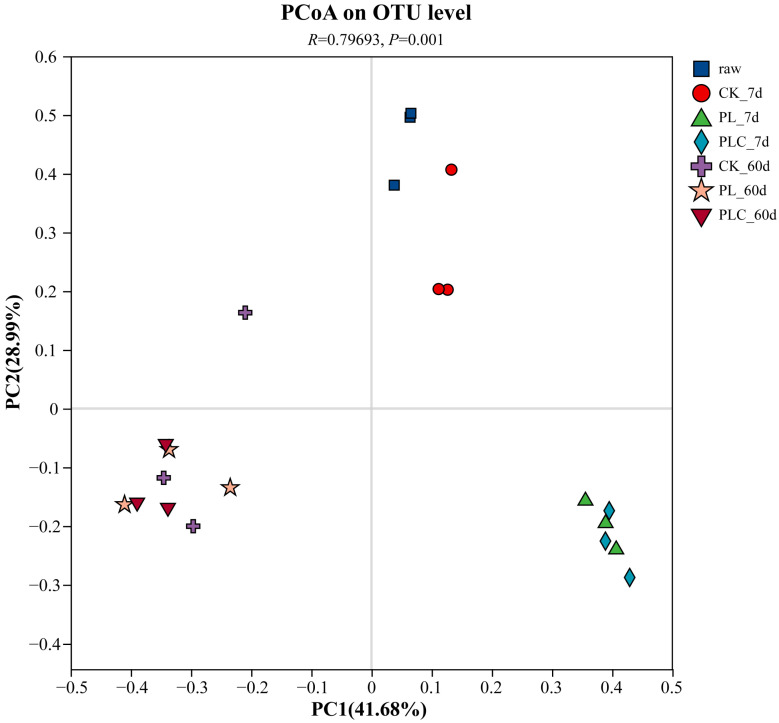
Analysis of bacterial diversity in Grape branch and leaf silage based on Principal coordinate Analysis (PCoA). Note: PL refers to the addition of *L. plantarum* (5 × 10^6^ CFU g^−1^ fresh weight), and PLC refers to the addition of *L. plantarum* (5 × 10^6^ CFU·g^−1^ fresh weight) and cellulase (0.3% fresh weight, enzyme activity ≥ 5000 U·g^−1^). Raw, raw material.

Principal coordinates analysis (PCoA) at the OTU level revealed significant shifts in bacterial community structure under different treatments and fermentation times (*R* = 0.7969, *p* = 0.001; Figure 3).

### 3.5. Analysis of Relative Abundance of Microbial Communities in Silage of Grape Branches and Leaves

**Figure 4:**
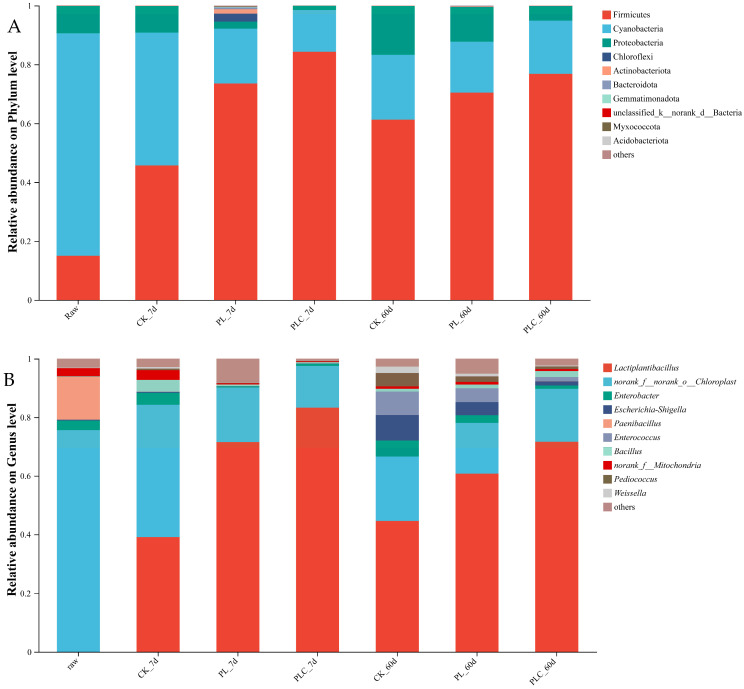
(**A**) The microbial community structure of grape branch and leaf silage at the gate level. (**B**) Microbial community structure belonging to horizontal silage of grape branches and leaves. Note: PL is the addition amount of *L. plantarum* (5 × 10^6^ CFU·g^−1^ fresh weight), and PLC is the addition amount of *L. plantarum* (5 × 10^6^ CFU·g^−1^ fresh weight) and cellulase (0.3% fresh weight, enzyme activity ≥ 5000 U·g^−1^).

Figure 4A depicts the relative abundance of microbial communities at the phylum level in raw materials and each post-fermentation group. In raw materials, Cyanobacteria exhibited the highest relative abundance (75.60%). Across all post-fermentation treatments (7CK, 7PL, 7PLC, 60CK, 60PL, 60PLC), Firmicutes was the dominant phylum (45.69–84.32%), laying a taxonomic foundation for lactic acid accumulation during silage.

At the genus level (Figure 4B), *Norank_f_norank_o_Chloroplast* had the highest relative abundance in raw materials (75.60%), while functional bacteria such as *Lactiplantibacillus* were extremely low. On the 7th day of fermentation, *Lactiplantibacillus* was the dominant bacterium in each group (39.08–83.28%). By the 60th day, *Lactiplantibacillus* remained the absolute dominant genus in 60PL and 60PLC (60.76–71.83%), much higher than that in 60CK (44.57%).

**Figure 5:**
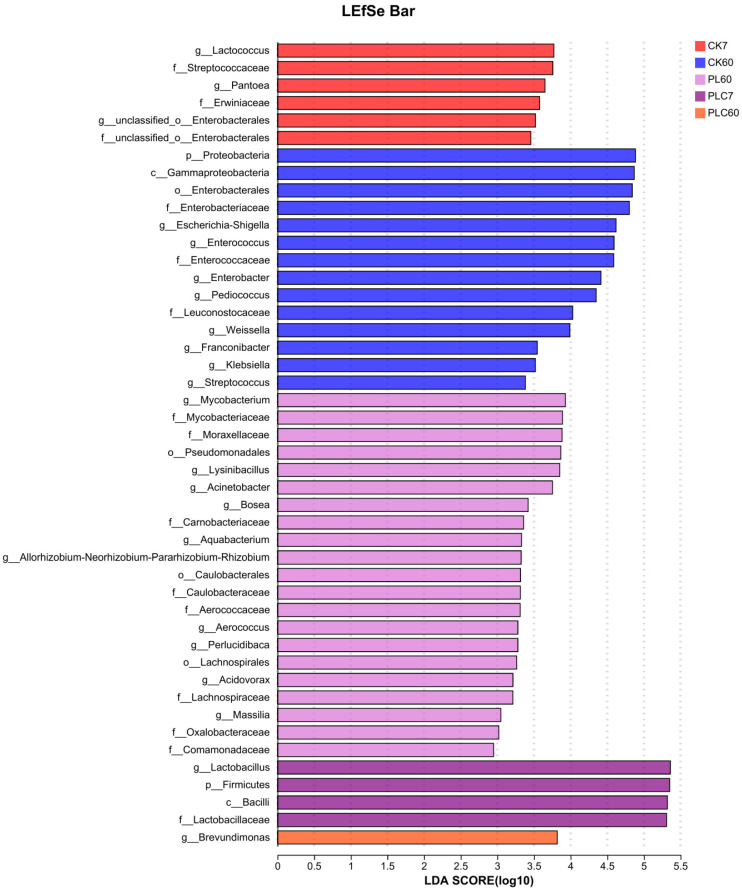
LEfSe analysis of Grape branch and leaf silage. Note: PL represents the dosage of *L. plantarum* (5 × 10^6^ CFU·g^−1^ fresh weight), and PLC represents the dosage of *L. plantarum* (5 × 10^6^ CFU·g^−1^ fresh weight) combined with cellulase (0.3% of fresh weight, enzyme activity ≥ 5000 U·g^−1^).

LEfSe was performed to further explore variations in the bacterial communities among the groups (Figure 5). On the 7th day, related CK7 group, taxa such as *Lactococcus* and *Pantoea* exhibited higher LDA scores, being the distinct bacterial taxa. On the 60th day, related CK60 group, *Escherichia-Shigella*, *Enterococcus*, *Enterobacter*, *Pediococcus*, *Weissella*, *Klebsiella*, and *Streptococcus* showed significant differences with high LDA scores. For the 60th day related PL60 group, *Streptococcus* and *Mycobacterium* were the taxa with prominent differences. In the 7th day related PLC7 group, *Lactiplantibacillus* had relatively high LDA scores, being the distinct taxon. In the 60th day related PLC60 group, *Brevundimonas* was the taxon with a significant difference, indicated by its high LDA score.

### 3.6. Correlation Analysis of Microbial Communities and Fermentation Indicators in Silage of Grape Branches and Leaves

**Figure 6:**
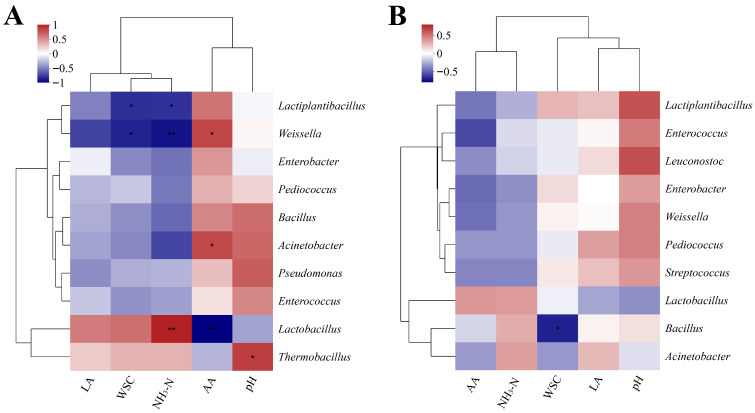
(**A**) Correlation analysis of microbial community (at the genus level) and fermentation characteristics on the 7th day of silage fermentation of grape branches and leaves; (**B**) Correlation analysis of microbial community (genus level) and fermentation characteristics on the 60th day of silage fermentation of grape branches and leaves. Correlation analysis of microbial communities (at the genus level) and fermentation characteristics on the 7th and 60th days of silage fermentation of grape branches and leaves. Note: NH_3_-N, ammoniacal nitrogen. AA, acetic Acid. LA, lactic acid. WSC, water-soluble carbohydrate. “*” indicates significant correlation (*p* < 0.05); “**” indicates highly significant correlation (*p* < 0.01).

*Spearman* correlation analysis was conducted on the dominant bacterial genera and silage fermentation indicators (LA, WSC, NH_3_-N, AA, pH) on the 7th day (Figure 6A) and day 60 (Figure 6B). On the 7th day, *Weissella* was significantly positively correlated with WSC (*R* = 0.58, *p* < 0.05) and extremely significantly positively correlated with NH_3_-N (*R* = 0.72, *p* < 0.01). *Lactiplantibacillus* was extremely significantly positively correlated with NH_3_-N (*R* = 0.80, *p* < 0.01) and AA (*R* = 0.75, *p* < 0.01). *Thermobacillus* was significantly positively correlated with pH (*R* = 0.60, *p* < 0.05).

On the 60th day, Bacillus was significantly negatively correlated with WSC (*R* = −0.55, *p* < 0.05). *Lactiplantibacillus* showed a strong positive correlation with pH (*R* = 0.70, *p* < 0.01). *Weissella* was significantly positively correlated with LA (*R* = 0.60, *p* < 0.05). These correlations indicate that different bacterial genera exert distinct regulatory effects on grape branch and leaf fermentation at different stages, with some genera closely associated with the accumulation of organic acids and the degradation of nutrients.

## 4. Discussion

### 4.1. Effects of Lactiplantibacillus plantarum and Cellulase on Nutritional Quality, Fermentation Traits, and Microbial Counts of Grape Branch and Leaf Silage

From the perspective of nutritional quality regulation, both inoculation treatments slowed DM loss, with the PLC treatment showing a more significant advantage during later stages. This difference can be attributed to the enzymatic hydrolysis by cellulase. Grape branches and leaves contain high proportions of structural carbohydrates (cellulose, hemicellulose). When *Lactiplantibacillus* is inoculated alone, its metabolic activity may decline due to insufficient soluble carbon sources. In the PLC treatment, however, cellulase continuously degrades structural carbohydrates, providing additional soluble substrates for *Lactiplantibacillus*. This prevents the decline in *Lactiplantibacillus* activity due to carbon shortage and reduces DM losses caused by ineffective microbial respiration and decomposition by miscellaneous bacteria [23]. The intergroup differences in CP reflect the regulatory effect of inoculation on proteolytic bacteria. *Lactiplantibacillus* inhibits these bacteria in the early stage [24], while in the later stage, a slight decrease in CP occurs as *Lactiplantibacillus* numbers decline and minor proteolysis by miscellaneous bacteria resumes [25].

The decreases in NDF and ADF are due to the enhanced decomposition by *Lactiplantibacillus*, cellulase, and other beneficial microorganisms utilizing fiber decomposition products during silage [26,27], which is consistent with our results. WSC content decreased continuously in all three treatments. Although not significantly different among treatments, WSC in the PLC treatment was initially slightly higher, due to the supplementary effect of cellulase hydrolyzing structural carbohydrates into WSC [28]. The subsequent decrease in WSC was a result of its conversion into fermentation products by *Lactiplantibacillus* [29].

Regarding fermentation characteristics, pH decreased continuously in all treatments, with the PLC treatment maintaining the significantly lowest pH in the later stages. This was due to the continuous accumulation of LA and AA produced by *Lactiplantibacillus*, indicating more stable acid production under the synergistic action of *Lactiplantibacillus* and cellulase [30]. A lower pH is more conducive to inhibiting miscellaneous bacteria. The content of LA first increased and then decreased. The reason was that in the early stage, the carbon source was abundant, which was conducive to the large-scale synthesis of LA by *Lactiplantibacillus*. Subsequently, the carbon source decreased, and the activity of *Lactiplantibacillus* decreased. In the later stage, secondary microorganisms partially converted LA into other metabolites [29]. The fluctuating AA content, slightly higher in the PLC treatment at later stages, might be related to a shift toward heterofermentative metabolism in the microbial community during later silage stages [31]. The NH_3_-N/TN ratio was lower in the PL and PLC treatments than in CK during the early stage, indicating that exogenous *Lactiplantibacillus* inhibited the deamination activity of proteolytic bacteria through rapid acid production, reducing protein conversion to NH_3_-N. Cellulase addition further reduces the alternative utilization of protein by *Lactiplantibacillus* via carbon source supplementation, decreasing nitrogen loss from metabolic pathways and thereby protecting protein [32].

Structural and functional changes in microbial communities are the fundamental drivers of these regulatory effects. Although LAB counts decreased in all treatments, the PL treatment maintained significantly higher counts than the PLC treatment in later stages. This is likely because the initial colonization of *Lactiplantibacillus* was more stable in the PL treatment, allowing it to sustain its population through competition even as carbon sources diminished. In the PLC treatment, the carbon source supplementation from cellulase also promoted the growth of other functional microorganisms, leading to slightly stronger interspecific competition for *Lactiplantibacillus* and consequently slightly lower counts. The PLC treatment demonstrated more prominent inhibition of yeast, aerobic bacteria, and molds, primarily due to the low-pH microenvironment maintained by the synergy of enzymes and bacteria [33]. The acidic environment effectively restricts the reproduction of aerobic, harmful microorganisms. Yeasts, as facultative anaerobes, also struggle to proliferate extensively under sustained acidic conditions. This environmental screening reduces nutrient consumption by harmful microorganisms, ensuring silage stability in the later stages [34].

In conclusion, both PL and PLC treatments improved the quality of grape branch and leaf silage by regulating the microbial community and material metabolism. However, the PLC treatment, benefiting from the synergy between cellulase and *L. plantarum*, demonstrated superior performance in fiber degradation, acid production stability, and inhibition of harmful microorganisms, providing scientific and technical support for the efficient utilization of grape branches and leaves as unconventional feed resources [35].

### 4.2. Effects of Lactiplantibacillus plantarum and Cellulase on Bacterial Community Composition of Grape Branch and Leaf Silage Fermentation

At 7 days of fermentation, no significant differences were observed in microbial diversity indices among the CK, PL, and PLC treatments, indicating similar species richness and diversity initially. Alpha diversity indices reflect the richness and evenness of the bacterial community in silage [36], and the natural microbiota on grape branches and leaves appeared sufficient to meet microbial requirements in the early fermentation stage [37]. *Lactiplantibacillus* became the dominant genus in the PL and PLC treatments. Its high abundance at the initial stage allowed rapid acid production, lowering pH, inhibiting harmful microorganisms, and improving silage quality. By day 60, the higher Simpson index in the PLC treatment suggested that this treatment was more conducive to maintaining microbial community diversity in the later stages, contributing to system stability.

In terms of community composition, *Cyanobacteria* were relatively abundant in the raw materials. After fermentation, *Firmicutes* became the dominant phylum in all treatments. The LAB within this phylum are well-adapted to the anaerobic and acidic silage environment, proliferating extensively to dominate the fermentation [38], thereby inhibiting aerobic *Cyanobacteria* and other treatments, which demonstrates the reshaping effect of silage fermentation on the microbial community [39]. *Successional* differences in dominant bacteria were observed among treatments: *Streptococcus* was relatively more abundant in the PLC treatment at 7 days. By day 60, *Proteobacteria* dominated in the CK treatment, *Lactiplantibacillus* in the PL treatment, and *Brevundimonas* in the PLC treatment. In the PL treatment, the sustained colonization of *Lactiplantibacillus* ensured continuous lactic acid production and a low pH environment. The CK treatment, lacking exogenous inoculants, showed relative enrichment of aerobic or facultative miscellaneous bacteria. The PLC treatment, under the synergy of cellulase and *L. plantarum*, developed a unique dominant bacterium, *Brevundimonas*, participating in the later fermentation.

From the correlation analysis, genera like *Weissella*, *Pediococcus*, and *Acinetobacter* were negatively correlated with pH and positively correlated with AA and NH_3_-N/TN, suggesting their involvement in acid production or protein decomposition, thereby influencing the fermentation environment and nitrogen form [40,41,42]. The direct correlation between *Lactiplantibacillus* and these indicators was relatively weak. *Lactiplantibacillus* mainly provides a fundamental guarantee for silage quality by rapidly producing acid in the early stage of fermentation to establish a low pH anaerobic environment, thereby regulating the relationship between other genera and fermentation indicators [43]. In conclusion, fermentation time and inoculation treatment jointly drove the succession and functional differentiation of the microbial community in grape branch and leaf silage. The PLC treatment showed particular potential in regulating silage quality by maintaining community diversity in the later stages and shaping a unique dominant bacterial genus. Our study benefits grape branch and leaf utilization and unconventional silage optimization. Co-treating grape waste with *L. plantarum* and cellulase (PLC) turns it into high-quality, nutrient-rich silage, easing forage shortages and cutting costs. The 30–60-day optimal silage period avoids quality loss, boosts grape industry circulation, and supports sustainable livestock farming.

## 5. Conclusions

This study verifies the core hypothesis that synergistic inoculation of *L. plantarum* and cellulase improves grape branch and leaf silage quality. The addition of *L. plantarum* and cellulase during the silage of grape branches and leaves greatly improved the silage quality. Compared with the CK and the PL, this combination can accelerate the reduction of pH value, effectively inhibit the growth of harmful microorganisms such as molds and yeasts, and significantly reduce the content of NDF and ADF. These improvements directly boost the nutritional availability and functional suitability of the silage as unconventional feed. In conclusion, the combined application of *L. plantarum* and cellulase is a practical strategy to convert low-value grape by-products into high-quality feed, supporting feed resource diversification and sustainable livestock production.

## Figures and Tables

**Table 1:** Nutritional Composition and Microbial Quantity of Grape branch and leaf silage Raw materials.

Index	Content
DM/%	31.5 ± 0.83
CP/%DM	18.9 ± 0.26
NDF/%DM	52.4 ± 0.97
ADF/%DM	27.1 ± 0.43
EE/%DM	5.1 ± 0.12
WSC/%DM	7.1 ± 0.21
LAB/logCFU·g^−1^ FM	7.9 ± 0.08
Yeast/logCFU·g^−1^ FM	4.1 ± 0.05
Mold/logCFU·g^−1^ FM	2.6 ± 0.06
AB/logCFU·g^−1^ FM	8.4 ± 0.02

Footnote: DM, dry matter; CP, crude protein; NDF, neutral detergent fiber; ADF, acid detergent fiber; EE, ether extract; WSC, water-soluble carbohydrates; LAB, lactic acid bacteria; AB, aerobic bacteria; FM, fresh weight. Data are presented as the means ± standard error.

**Table 2:** The changes in nutritional quality during the fermentation process of grape branch and leaf silage.

Index	Time	Groups			*p*-Value		
		CK	PL	PLC	Groups	Time	G × T
DM%	7 d	36.1 ± 0.82 Aa	36.6 ± 1.14 Aa	37.3 ± 1.82 Aa	0.0001	0.0001	0.0117
	15 d	34.3 ± 0.52 Bb	35.3 ± 0.29 Aab	36.7 ± 0.88 ABa			
	30 d	31.3 ± 1.18 Cc	33.0 ± 0.14 Bb	35.7 ± 0.08 Ba			
	60 d	28.5 ± 0.39 Dc	30.8 ± 0.63 Cb	35.7 ± 0.08 Ca			
CP% DM	7 d	15.8 ± 0.04 Cc	17.9 ± 0.06 Ab	18.6 ± 0.06 Aa	0.0001	0.0001	0.0001
	15 d	16.0 ± 0.08 Cb	17.9 ± 0.06 Aa	17.8 ± 0.13 Ba			
	30 d	16.5 ± 0.02 Bb	16.4 ± 0.17 Bb	16.7 ± 0.01 Ca			
	60 d	17.0 ± 0.03 Aa	16.6 ± 0.31 Bb	16.4 ± 0.36 Db			
EE% DM	7 d	3.5 ± 0.31 Ba	4.1 ± 0.13 Ca	3.9 ± 0.29 Ca	0.8676	0.0001	0.4391
	15 d	6.2 ± 0.08 Aa	6.4 ± 0.53 Aa	6.2 ± 0.19 Aa			
	30 d	6.0 ± 1.12 Aa	5.6 ± 0.62 Ba	5.5 ± 0.38 Ba			
	60 d	4.1 ± 0.21 Ba	4.0 ± 0.05 Ca	4.4 ± 0.27 Ca			
WSC% DM	7 d	7.5 ± 0.33 Ab	8.0 ± 0.27 Aab	8.4 ± 0.48 Aa	0.1624	0.0001	0.3974
	15 d	7.2 ± 0.63 Aa	7.3 ± 0.54 Aa	7.5 ± 0.48 Ba			
	30 d	5.9 ± 0.40 Ba	6.4 ± 0.43 Ba	6.4 ± 0.62 Ca			
	60 d	5.4 ± 0.27 Ba	5.0 ± 0.23 Ca	5.1 ± 0.50 Da			
NDF% DM	7 d	49.4 ± 0.33 Aa	48.8 ± 0.53 Aa	47.5 ± 0.38 Ab	0.0001	0.0001	0.7395
	15 d	48.5 ± 0.65 Ba	47.6 ± 0.41 Ba	46.4 ± 0.34 Bb			
	30 d	48.0 ± 0.64 Ba	46.6 ± 0.52 Cb	45.9 ± 0.15 BCb			
	60 d	47.0 ± 0.66 Ca	45.9 ± 0.91 Cb	45.4 ± 0.50 Cb			
ADF% DM	7 d	25.5 ± 0.58 Aa	23.2 ± 0.52 Ab	22.2 ± 0.70 Ac	0.0001	0.0001	0.3395
	15 d	23.7 ± 0.28 Ba	22.3 ± 0.75 Bb	20.3 ± 0.68 Ac			
	30 d	21.8 ± 0.35 Ca	20.8 ± 0.87 Ba	19.5 ± 0.15 Bb			
	60 d	20.6 ± 0.49 Da	19.1 ± 0.47 Cb	17.9 ± 0.64 Cc			

Footnote: CK in the above table represents the control group, PL indicates addition of *L. plantarum*, and PLC indicates addition of *L. plantarum* and cellulase. The following table is the same. The same indicator in the same column is marked with different capital letters (such as A, B, C, D), indicating that the difference in the number of fermentation days under the same treatment is statistically significant. Lowercase letters within the same line (such as a, b, c) indicate significant differences among groups on the day of fermentation. Data are expressed as mean ± standard error.

**Table 3:** The changes in the number of microorganisms during the fermentation process of grape branch and leaf silage.

Index	Time	Groups			*p*-Value		
		CK	PL	PLC	Groups	Time	G × T
LAB (logCFU·g^−1^ FM)	7 d	7.4 ± 0.07 Aa	7.2 ± 0.07 Aa	7.2 ± 0.17 Aa	0.0001	0.0001	0.0140
	15 d	6.8 ± 0.27 Ba	6.9 ± 0.02 Ba	6.7 ± 0.22 Ba			
	30 d	6.4 ± 0.28 Ca	6.6 ± 0.37 Ba	5.8 ± 0.07 Cb			
	60 d	5.8 ± 0.05 Da	6.0 ± 0.18 Ca	5.2 ± 0.16 Db			
Yeast (logCFU·g^−1^ FM)	7 d	4.1 ± 0.10 Aa	3.9 ± 0.08 Aab	3.8 ± 0.08 Ab	0.0001	0.0001	0.0759
	15 d	3.9 ± 0.05 Aa	3.5 ± 0.03 Bb	3.6 ± 0.10 Bb			
	30 d	3.7 ± 0.08 Ba	3.4 ± 0.18 Bb	3.3 ± 0.11 Cb			
	60 d	3.2 ± 0.13 Ca	3.1 ± 0.03 Ca	3.0 ± 0.04 Da			
Mold (logCFU·g^−1^ FM)	7 d	2.2 ± 0.14 Ca	2.3 ± 0.06 Ba	2.2 ± 0.01 ABa	0.0002	0.0001	0.0291
	15 d	2.4 ± 0.06 Ca	2.1 ± 0.04 Ba	2.0 ± 0.01 Ba			
	30 d	2.9 ± 0.06 Ba	2.7 ± 0.01 Aab	2.4 ± 0.05 Ab			
	60 d	3.4 ± 0.52 Aa	3.0 ± 0.45 Aa	2.4 ± 0.15 Ab			
Aerobic bacteria (logCFU·g^−1^ FM)	7 d	8.1 ± 0.06 Aa	8.0 ± 0.05 Aa	7.7 ± 0.05 Ab	0.0001	0.0001	0.2575
	15 d	7.7 ± 0.16 Ba	7.6 ± 0.16 Ba	7.6 ± 0.21 Aa			
	30 d	7.1 ± 0.10 Ca	7.0 ± 0.15 Ca	6.9 ± 0.15 Ba			
	60 d	6.4 ± 0.23 Da	6.5 ± 0.10 Da	6.3 ± 0.06 Ca			

Footnote: CK in the above table represents the control group, PL indicates addition of *L. plantarum*, and PLC indicates addition of *L. plantarum* and cellulase. The following table is the same. The same indicator in the same column is marked with different capital letters (such as A, B, C, D), indicating that the difference in the number of fermentation days under the same treatment is statistically significant. Lowercase letters within the same line (such as a, b) indicate significant differences among groups on the day of fermentation. Data are expressed as mean ± standard error.

## Data Availability

The data presented in this study are available upon request from the first author. Data supporting the bacterial community in the Sequence Read Archive (SRA) can be found at the National Center for Biotechnology Information (NCBI). SRA number: PRJNA1348612.

## Abbreviations

The following abbreviations are used in this manuscript:

ADF	Acid detergent fiber
CP	Crude protein
EE	Ether extract
DM	Dry matter
WSC	Water-soluble carbohydrate
LAB	Lactic acid bacteria
PL	*Lactiplantibacillus plantarum*
PLC	*Lactiplantibacillus plantarum* and cellulase
NDF	Neutral detergent fiber
NH_3_-N	Ammoniacal nitrogen
PA	Propionic acid
AA	Acetic acid
BA	Butyric acid
LA	Lactic acid
